# Accurate Sample Time Reconstruction of Inertial FIFO Data †

**DOI:** 10.3390/s17122894

**Published:** 2017-12-13

**Authors:** Sebastian Stieber, Rainer Dorsch, Christian Haubelt

**Affiliations:** 1 Department of Applied Microelectronics and Computer Engineering, University of Rostock, 18109 Rostock, Germany; christian.haubelt@uni-rostock.de; 2 BOSCH Sensortec GmbH, 72770 Reutlingen, Germany; rainer.dorsch@bosch-sensortec.com

**Keywords:** sensor nodes, synchronization, Hifi sensors, sensor time, FIFO, MEMS, raspberry pi

## Abstract

In the context of modern cyber-physical systems, the accuracy of underlying sensor data plays an increasingly important role in sensor data fusion and feature extraction. The raw events of multiple sensors have to be aligned in time to enable high quality sensor fusion results. However, the growing number of simultaneously connected sensor devices make the energy saving data acquisition and processing more and more difficult. Hence, most of the modern sensors offer a first-in-first-out (FIFO) interface to store multiple data samples and to relax timing constraints, when handling multiple sensor devices. However, using the FIFO interface increases the negative influence of individual clock drifts—introduced by fabrication inaccuracies, temperature changes and wear-out effects—onto the sampling data reconstruction. Furthermore, additional timing offset errors due to communication and software latencies increases with a growing number of sensor devices. In this article, we present an approach for an accurate sample time reconstruction independent of the actual clock drift with the help of an internal sensor timer. Such timers are already available in modern sensors, manufactured in micro-electromechanical systems (MEMS) technology. The presented approach focuses on calculating accurate time stamps using the sensor FIFO interface in a forward-only processing manner as a robust and energy saving solution. The proposed algorithm is able to lower the overall standard deviation of reconstructed sampling periods below 40 μs, while run-time savings of up to 42% are achieved, compared to single sample acquisition.

## 1. Introduction

Applications for virtual and augmented reality place exacting requirements to sensor data, especially in case of movement tracking. In our previous work, we identified the negative influence of inaccurate time stamps onto subsequent processing algorithms [1]. However, in the areas including fitness tracking, activity recognition, gesture detection and many more, the fusion of multiple sensor data streams forms the basis for highly sophisticated feature extraction algorithms. The increasing availability of versatile types of sensor data is made possible by micro-electromechanical systems (MEMS). MEMS have become the state-of-the-art technology for extremely small, robust and energy efficient sensor devices. They combine mechanical elements, measuring physical quantities, with traditional integrated circuit components on a single chip [2]. Modern inertial MEMS sensors measure, among others, acceleration and rotation several thousand times per second with a high resolution. They are built into more and more everyday objects, such as smartphones, fitness trackers, game controllers, VR devices, jewelery, clothes and many more [3,4].

With the increasing number of sensor devices, as well as sensor data types, the acquisition of the sensor data samples becomes time and energy consuming, which is undesirable on low power wearable devices. The sleep time of the hosts application processor gets interrupted by a variety of sensor devices to offer new sampled data. Shojafar et al. also proposes an adaption of the communication rate to lower the overall energy consumption [5]. Most modern sensors support the data acquisition one-by-one from dedicated sensor data registers and additionally the storage of multiple sensor samples in a first-in-first-out (FIFO) structure [6]. Using the FIFO interface can increase the sleep time of the application processor by reducing host-sensor communication [7]. Furthermore, timing constraints, when fetching data from multiple sensor devices, are relaxed and data loss can be avoided.

**Figure 1:**
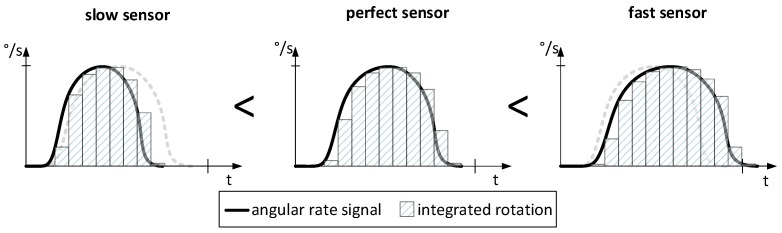
Exemplary illustration of the integration error due to relative sensor clock drift for a slow, perfect and fast running sensor clock, respectively.

However, some negative effects, e.g., clock drifts and communication delays, are amplified by the usage of the FIFO interface. The process of reading a greater amount of data takes more time and a reconstruction of the sampling times of the data samples in the FIFO stream is necessary. For example, considering the sensor data processing of a virtual or augmented reality (VR/AR) application, the integration of sampled data of a FIFO stream without consideration of the relative drift between the time bases of the host and the sensor device will introduce a severe error in the orientation vector. Figure 1 shows the influence of the relative clock drift on the integration result. If the sensor clock is running slower or faster than expected, the sensor will create less, respectively more samples in the same time. The resulting total error in distance and rotation increases with time and strength of the movement. Also, to allow a high quality multi sensor data fusion, the input data streams have to be synchronized in time. Another use case of an accurate synchronization is shown in [8]. Many routing algorithms in wireless sensor networks require a synchronized time base to optimize routing decisions.

Our goal is to create an algorithm able to calculate accurate time stamps of sampling events on the host device targeting the compensation of drift and offset errors of multiple sensor streams. As a result, the reconstructed data of different sensors will be aligned in time. Furthermore, batch processing of sensor data fetched from FIFO interfaces must be possible in order to lower the overall energy consumption. In this study, we mainly focus on bus-driven communication between host and sensor devices.

In this article, we present the accurate sample time reconstruction (ASTR) as an approach to calculate precise sampling time stamps using batched high frequency FIFO data of modern inertial MEMS sensors. Our proposed algorithm relies on the fact that the sensor doesn’t provide time stamps for each sample but instead the sensor time for the FIFO readout; the age of each sensor sample can be determined based on a fixed relation between the sampling time, a configurable output data rate and the current timer value of the sensor device. Hence, each sensor device must be able to offer the current sensor time accurately on a host read access. By fetching multiple of those sensor times, a comparison with the local host times allows the calculation of relative time based deviations between the sensor devices and the host. The sensor time stamp can also be added to the end of the fetched FIFO data, enabling the combination of data acquisition and time stamp synchronization.

In the next Section, we will give an overview about existing works in the field of sensor data synchronization and do a classification of the ASTR and other algorithms. Section 3 explains in detail the background of the targeted synchronization issues, as well as an exemplary FIFO interface and sensor timer. Additionally, a basic signal reconstruction algorithm is shown, that can be used to reconstruct the FIFO stream data. In Section 4, the ASTR is derived to address drift and offset errors of the basic reconstruction approach. Using ASTR, we are able to keep the standard deviation of the reconstructed sampling periods below 40 μs. Furthermore, the processor utilization could be lowered by 42% in the performed experiments, compared to a single sample acquisition approach. The detailed results can be read in Section 5. A short conclusion is given in Section 6.

## 2. Related Work

There have been many publications in the last years, dealing with the problem of accurate synchronization of sensor events. The existing works can mainly be classified into two major groups. Most of the available publications deal with the topic of clock synchronization. That means, the internal clock of the sensor devices can be adjusted in their value, frequency or both. That allows a direct synchronization of the wired or wireless reachable sensor devices in phase and frequency. The data acquisition itself happens without further modifications and fetched sensor time stamps equals the host time base.

Cristian’s Algorithm [9] serves as the basis for many clock synchronization algorithms. A message is sent from the sensor node to the host, which acts as the time server and returns the timing information immediately. Then the sensor node sets its clock to the fetched time increased by the half of the total communication time. The accuracy of this approach varies with the amount of communication latency and jitter. The Berkeley Algorithm [10] uses the same mechanism, but here the host issues the time measurements repeatedly. The averaged time difference is used afterwards to synchronize the sensor clocks. Elson et al. defined the Reference Broadcast Synchronization Scheme (RBS) [11]. The idea of this approach is to exchange the timing information of each sensor node with its neighbors in a wireless sensor network upon receiving a synchronization pulse. With the time stamps of the other devices, a device’s own clock should be adjusted. In [12], Ganeriwal et al. proposed the Timing-Sync Protocol for Sensor Networks (TPSN), where the synchronization happens in a hierarchical manner. In a level discovery phase, the available sensor nodes are structured in multiple hierarchical levels with only one root node. In the synchronization phase, each level synchronizes using a two way message exchange between pairs of nodes, starting with a sync message from the root node. A more detailed overview about the protocol described, as well as additional clock synchronization approaches, are given in the survey of Sivrikaya et al. [13].

Although clock synchronization is able to compensate for the error introduced by the relative clock drifts, there are many sensor devices that can’t adjust their internal clock. To synchronize the data of those sensors, the fetched sensor data time stamps have to be adjusted at the host in order to align different time bases. This mechanism is called time stamp synchronization.

The idea of time synchronization without modifying the clocks of the distributed sensor devices was presented by Römer and Elson in [14,15]. Among other ideas, they considered a communication time stamp translation mechanism between several nodes. Therefore, a time stamp is converted to Coordinated Universal Time (UTC), while it is transferred between different sensor nodes. The message delay is measured with a two way message exchange scheme, similar to TPSN, and subtracted from the receiver time, when the message arrived. Finally, lower and upper bounds of a time stamp estimation are calculated in the target device. Donovan et al. proposed a method for sensor synchronization by centralized triggering the measurements in a network with deterministic access times [16]. By starting the measurements at the same time, a synchronization of the resulting time stamps can be omitted. However, while this can be done with some kinds of sensors, such as magnetic field sensors, it introduces a high communication overhead. Furthermore, it is not applicable for continuously sensing devices, such as inertial sensors, especially gyroscopes. Olson presented another time stamp synchronization method in [17] without changing the sensor clock. This method runs totally passive at the host. While a sensor node sends its data and local time stamp to the host periodically, multiple data sets are used by the algorithm to estimate the minimum latency of the system and to correct the sensor time stamp.

Our approach can also be classified as a time stamp synchronization approach, which is compatible with sensor devices that can’t adjust their local clock. While the main idea of a passive synchronization, which is completely done on the host, operates nearly to the solution of Olson, in our approach, the host initiates the read access to the sensor devices. The process of synchronization is fully combined with the data acquisition and by using the correlation between the sensor timer and the sampling times, the exact age of the fetched sensor data can easily be determined. In contrast to existing approaches, we focus on stream processing the sensor data fetched from the FIFO interface. Hence, the approach includes the reconstruction of the single sensor samples from the FIFO stream as well as their drift and offset corrected sampling time stamps. In addition to our previous work in [1], the ASTR is refined by considering the bandwidth of different underlying communication systems in order to compensate timing offset errors between multiple sensor devices. Furthermore, the influence of communication jitter on the measured amount of relative clock drift between the host and the sensor devices is reduced by using low-pass filter mechanisms. To prove our simulated findings in [1], we performed experiments with real sensors and improved the metric for a better comparability of the results.

## 3. Background and Problem Statement

While most modern mobile devices and wearables are equipped with a variety of sensors, more and more people are using applications that are based on the gathered sensor data. However, they are expecting highly accurate results, e.g., from activity recognition, step counting, heart rate measurements, indoor navigation and more. Sensor fusion plays a fundamental role in achieving those highly accurate results. Via sensor fusion, the information of multiple sensors gets combined to either improve the measured signal or to create a new logical sensor output [18].

The synchronicity of the different sensor signals is of major importance, as, for example, shifted or stretched signals would blur sensed events and therefore distort subsequent feature extraction algorithms. Due to fabrication variances, wear-out effects and temperature fluctuations, the clocks of the sensor devices can drift and influence the sample timing. Also, due to varying communication latencies, the read out sensor data is influenced by individual jitters and offsets.

**Figure 2:**
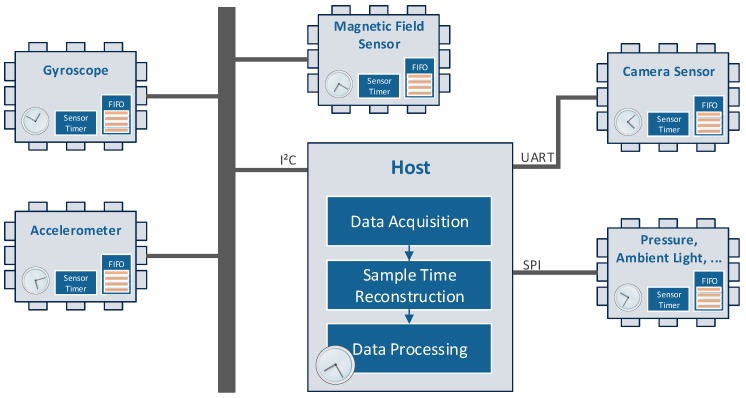
Architecture: sensor devices are directly connected to the host. They support a sensor timer and a first-in-first-out (FIFO) storage. Due to drift effects, each system runs on a different timebase.

Figure 2 shows the general architecture, which is used in the majority of sensor equipped devices. One or more sensor devices are connected to a host system. The host can be the application processor or co-processor of the mobile device. Additionally, in some devices, the sensors are connected to a sensor hub, which is usually performing the sensor data acquisition and sensor data fusion at a lower energy consumption level than the application processor. In this case, a hierarchical concatenation of the synchronization would be possible. To improve the sensor data fusion results, the raw input signals shall be synchronized. In particular, frequency drifts and offsets should be removed.

To perform our time stamp reconstruction approach, the sensor devices should offer a free-running sensor timer value. Moreover, to lower the energy consumption of the whole system, using the FIFO interface of the sensor devices is recommended.

### 3.1. FIFO Interface

**Figure 3:**
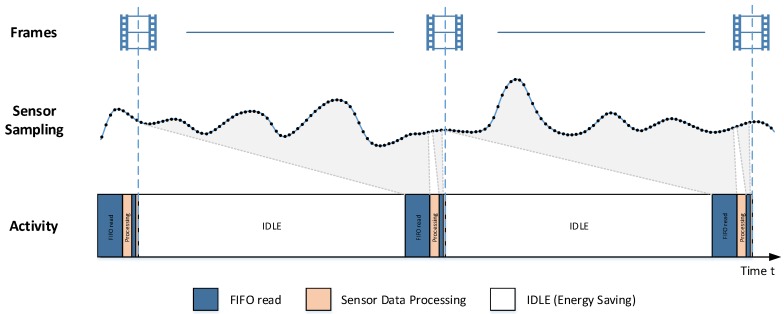
Using the FIFO interface of the sensor can lower the power consumption and avoid data loss. By double-reading the FIFO content, it is also applicable on low latency requirements.

The FIFO interface is a state-of-the-art storage feature in modern high data rate sensors, as e.g., inertial measurement units (IMUs). The measured sensor samples are stored in a ring buffer data structure and can be read out by the host in chronological order. As the FIFO interface allows the acquisition of multiple sensor samples at once, the host sensor communication happens less frequent and the host can stay longer in energy saving power modes. Additionally, data loss is avoided, as the host can read all available sensor data since the last FIFO access, as long as a FIFO storage overflow is avoided. It is obvious that compared to single frame readout, the total communication latency can be higher when reading many frames at once. This problem can be solved by reading the FIFO content twice. First, a bigger chunk of data is read a short time before the next upper layer event has to be computed and, just in time, a small number of sensor frames are read from FIFO, enabling low latency behavior. In Figure 3, the exemplary usage of this mechanism is shown in the context of a VR/AR application. While the latest VR and AR devices work with frame rates between 90 Hz and 120 Hz, there are still dozens of inertial sensor frames measured between two rendered pictures.

### 3.2. Sensor Timer

The sensor timer is an essential requirement of the ASTR. The host should be able to gather the latest timing information from the sensor devices to perform the time stamp reconstruction algorithm that is described in Section 4. The reconstruction algorithm works with any kind of sensor timer, as long as the sampling times are related to this timer. For example, in [6,19], the sensor timer is implemented as a 24-bit wide, free-running counter, which has a granularity of 39.0625 μs. The timer is strictly synchronized with the sampling times of the measurement unit. As a result, a new set of data is stored, whenever a specific bit of the sensor timer toggles [20]. Table 1 shows the relationship between the sensor timer and the configurable sensor data rates. For example, if the sensor data rate is configured to run at 100 Hz, the corresponding bit *m* would be the eighth bit of the sensor timer.

Usually, the latest sensor timer value can be read by direct register access. Additionally, in [6], it is appended to the end of the FIFO content, whenever the FIFO interface is over-read by the host device.

### 3.3. Basic Signal Reconstruction

When gathering the sensor data efficiently by using the FIFO interface, the host has to reconstruct the sensor signal and to calculate time stamps $t_{n}$ for all sensor samples in the FIFO stream (n∈N, $0\leq n\leq {\#}_{samples}$). They depend on the configurable sampling period $period_{sensor}$ and the initial time stamp $t_{0}$, which describes the sample time of the first sensor sample in the FIFO stream. Based on the resulting time stamps the samples of different sensors can be synchronized and afterwards used by sensor fusion algorithms. A basic but often used implementation of a FIFO based forward-only reconstruction algorithm is shown in Equation (1), which is calculated for each available sensor device individually. A forward-only processing system parses the FIFO stream from the beginning and does not allow modifications of already processed items. This behavior can reduce the latency for further processing steps, based on single sensor samples.

(1) $$t_{n}=t_{0}+n\cdot period_{sensor}$$

The readout of the FIFO stream by the host can be triggered by a fill level interrupt of the sensor device. Once the FIFO storage exceeds a configurable amount of data, an interrupt is issued and the host starts reading the expected amount of data from the FIFO interface. At the same time, the host stores the time stamp $t_{lvl}$ of the interrupt, as this time nearly matches the sampling time of the last sample in the FIFO stream. Using this timing information, the initial reconstruction time stamp $t_{0}$ of the current FIFO stream can be calculated by Equation (2).

(2) $$t_{0}=t_{lvl-1}+period_{sensor}$$

The time stamp $t_{lvl-1}$ describes the sampling time of the last sample of the previously processed FIFO chunk. Thus, to calculate the time stamp of each sample of the FIFO stream, both equations can be combined to Equation (3).

(3) $$t_{n}=t_{lvl-1}+\left(n+1\right)\cdot period_{sensor}$$

**Figure 4:**
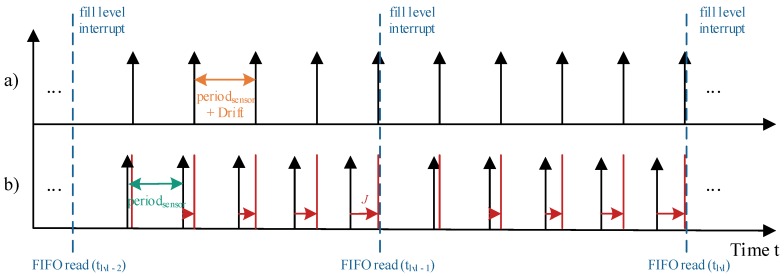
Exemplary result of basic time stamp reconstruction algorithm, where (**a**) represents the real samplings affected by a frequency deviation (drift) and (**b**) shows the reconstructed sampling times, highlighting the resulting Jitter *J*.

Since there is no information considered about the internal timing of the sensor system, $period_{sensor}$ is the ideal period of time between two measured samples, fitting the sensor configuration. While there are only slight frequency deviations between sensor and host system, this basic time stamp reconstruction algorithm achieves good results. However, with increasing the deviation of the frequencies, a rising error results depending on the configured fill level and thus on the size of the FIFO stream. Figure 4 illustrates the relationship between the FIFO fill level and the jitter of the reconstructed signal. The resulting jitter *J* increases steadily within one FIFO stream chunk.

## 4. Accurate Sample Time Reconstruction

The described basic signal reconstruction algorithm enables an energy efficient sensor data reading via the FIFO interface. Nevertheless, the reconstructed signal contains clock drift dependent jitter and communication latencies. Due to this fact, the calculated time stamps are not well synchronized, especially when using multiple different sensor devices with individual frequency drifts and communication systems.

To resolve this problem

- the ratio between the time bases of host and sensor has to be taken into account, and
- the offset between sampling time and calculation of the time stamp has to be eliminated.

Each sensor system must be able to offer the current sensor time value instead of sample-related time stamps to enable the following time stamp reconstruction approach. Many modern sensors, e.g., [6,19], already support such sensor timers. Moreover, in these devices the latest sensor time value can be fetched together with the FIFO content, which allows a combined data acquisition and accurate time stamp calculation.

### 4.1. Drift Measurement

To calculate the ratio between the time bases of two devices, the host device can read the timing information of the sensor system at least two times, gathering the sensor time stamps $ST_{a-1}$ and $ST_{a}$ (a∈N). Accordingly, the local host timing information of both communication attempts is stored as $HT_{a-1}$ and $HT_{a}$. With those time stamps, the relative drift *D* can be easily calculated by Algorithm 1. In this example, the sensor timer values are 24-bit wide and have a granularity of 39.0625 μs, while the host time stamps are already given in μs. The first few lines of the algorithm handle a potential overflow of the sensor timer value and the others calculate the ratio between the time bases of the sensor system and the host.

**Algorithm 1:** Simple drift calculation. Sensor time stamps ST are given as 24 bit values, while the related host time stamps HT are already scaled in μs. 
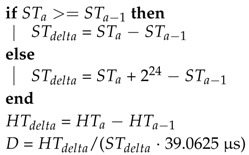


This procedure can be done asynchronously, in parallel to the sensor data reading. However, it is advantageous to combine the reading of the sensor time information and the data reading, as the sleep time of the host application processor can be further increased. For example, the sensor system in [6] allows the reading of sensor data together with the sensor timer value in a single burst access and also attaches the sensor timer value to the end of the FIFO stream, when it gets over-read. Repeating this calculation periodically increases the robustness against slow changing clock drifts. Furthermore, it is recommended to increase the timing window, which is used in the drift calculation, as it will reduce the influence of communication jitter (Algorithm 2). As an alternative, the drift value can be filtered with a low pass function to reduce higher frequent changes of the relative drift *D*.

**Algorithm 2:** More stable drift calculation. The influence of communication jitter is reduced by increasing the time window, using b∈N,b>1. 
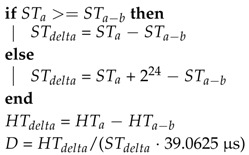


Once the relative drift between the host and sensor devices is calculated, it can be considered in the sensor time stamp reconstruction. The ideal sensor sampling period $period_{sensor}$ is weighted with the relative drift *D* to eliminate the temporal distortion between the different time bases. Equation (4) shows the calculation of drift corrected consecutive sensor sample time stamps $t_{n}$.

(4) $$t_{n}=t_{n-1}+D\cdot period_{sensor}$$

### 4.2. Sampling Time Offset Correction

**Figure 5:**
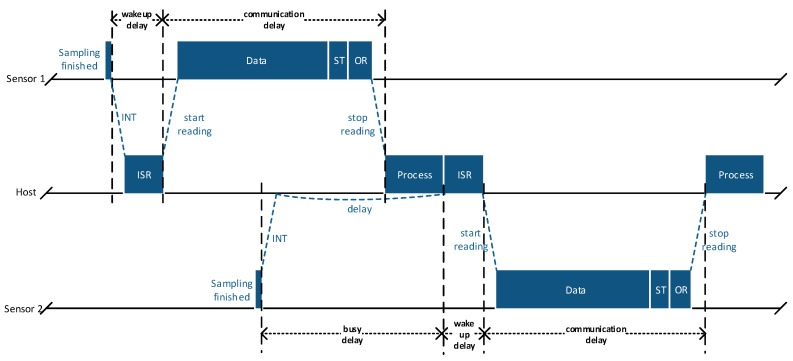
Exemplary communication between host and multiple sensor devices.

In addition to the different time bases of the host and the sensor devices, the gathered signal is affected by a temporal offset. Figure 5 shows an abstract timing diagram of a communication attempt between the host and two sensor devices. The host fetches many bytes from each FIFO interface including the sensor timer value, which is appended at the end of valid sensor data. However, to make sure getting the latest data within the FIFO stream, even when higher sample rates are used in the sensor devices, additional bytes are read by the host. Hence, the total FIFO data stream of each sensor device consists of multiple sensor samples, the sensor timer value (ST) and an invalid over-read section (OR). As the interrupts of the different sensors can trigger asynchronously, the readout of each FIFO stream can be delayed. It becomes clear that different sources of delay, e.g., software and communication delays, lead to noticeable timing offsets and jitter in the reconstructed sensor signal.

To address this issue, again the sensor timer can be used. As the sampling times of the sensor devices are derived from the corresponding timer, reading the latest sensor time value $ST_{a}$ gives information about the age of the last generated sensor sample. Using the synchronized sample mechanism, which is shown in Table 1, the age of the last sensor sample $t_{age}$ can be easily calculated by Equation (5). It is determined by the least significant bits (LSB) below the toggling sensor time bit *m*, reflecting the currently selected sensor data rate.

(5) $$t_{age}=\left(ST_{a}\;mod\;2^{m}\right)\cdot D\cdot 39.0625\;\mu s$$

The calculated $t_{age}$ covers the time between the sensor sampling and the read access of the host to the sensor timer value at the end of valid FIFO data. The remaining delay between the read access and the host time stamp creation $HT_{a}$ is decisively determined by the communication time $t_{OR}$ of the number of over-read bytes ${\#}_{overreadbytes}$. Especially with a fluctuating number of over-read bytes or sensor devices which are connected on different bus systems, the compensation of this delay becomes important. The calculation of $t_{OR}$ depends on the used data rate $t_{comm/byte}$ of the underlying bus architecture (refer Equation (6)), e.g., reading 100 bytes from a 10 MHz SPI bus takes about 80 μs, while reading the same amount of data from a 400 kHz fast-mode I2C bus takes more than 2 ms.

(6) $$t_{OR}={\#}_{overreadbytes}\cdot t_{comm/byte}$$

Equation (7) contains all the presented timing corrections and can directly be used to calculate accurate time stamps of each sensor sample within a FIFO stream in a forward-only processing system.

(7) $$t_{n}=\left(HT_{a}-t_{age}-t_{OR}\right)+\left(n+1\right)\cdot D\cdot period_{sensor}$$

The whole reconstruction of the FIFO stream including data samples and time stamps has a time complexity of O(n), as each input sample is touched once and the corresponding time stamp can be calculated by adding the drift-adapted sensor period to the previous sampling time stamp. The same applies for the space complexity. The number of created time stamps is equal to the number of processed sensor samples within the FIFO stream. However, the needed storage for the time stamps is noticeably smaller than the storage of the sensor data.

## 5. Experimental Results

To prove our ASTR approach, we conducted tests using two inertial sensor devices [6], as well as a simulation based test setup. Particularly, we looked at the sample-to-sample timing distance as a metric for the relative drift correction of each reconstructed signal. An overall small deviation of the reconstructed sampling periods, especially between the fetched FIFO streams, can be evaluated as successful drift correction.

### 5.1. Test Environment

Two inertial sensor devices of the type BMI160 [6] were connected to the SPI interface of a raspberry pi 2B platform, running standard raspbian linux. The sensors completely fulfill the requirements of the presented approach, as they provide a sensor timer as well as a FIFO interface. The algorithms for the basic and accurate reconstruction are implemented using Python 2.7 and executed directly on the raspberry pi.

**Figure 6:**
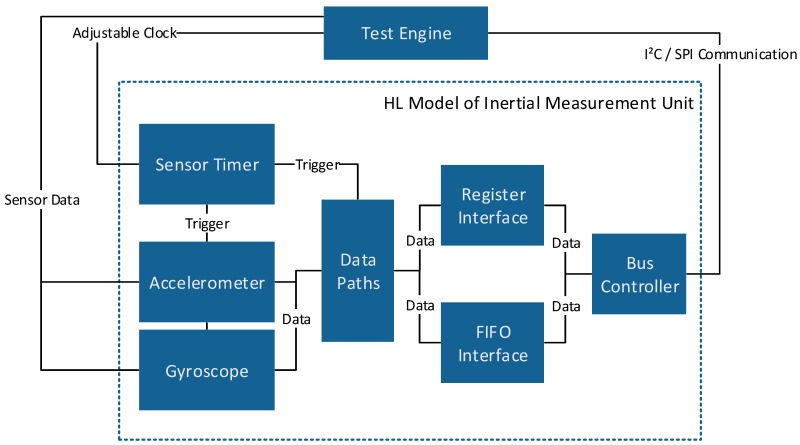
High level SystemC model of an inertial measurement unit.

To gather information about the synchronization robustness at higher relative drifts between host and sensor devices, the algorithms were tested in a simulation based environment, too. Therefore a virtual prototype, as depicted in Figure 6, of the above mentioned sensor devices was used.

The virtual prototype was implemented as a high-level SystemC [21] model. It contains the configurable sensor data paths of an acceleration and a gyroscopic sensor, as well as the FIFO interface and the sensor timer. Furthermore, the Serial Peripheral Interface (SPI) and Inter-Integrated Circuit (I2C) bus protocols are supported by the model. While changing the relative drift between host and sensor device clocks accurately is hard on real devices, the high-level model offers the opportunity to test the reconstruction algorithms with any desired time base distortion.

### 5.2. Results

**Figure 7:**
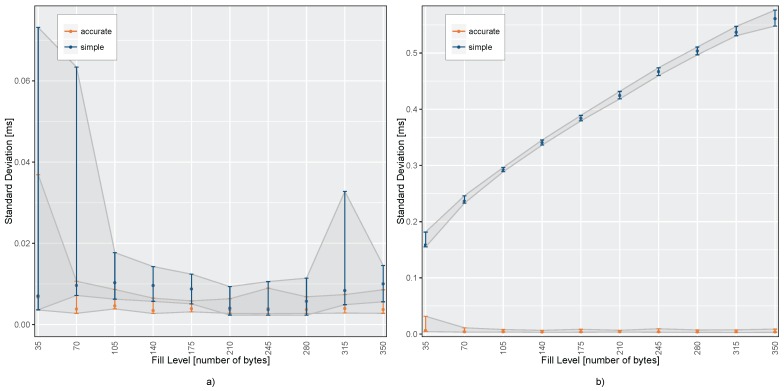
Comparison of resulting sample-to-sample distances for (**a**) sensor 1 with no measurable relative drift (D=0.0%) and (**b**) sensor 2 with a mean relative drift D=1.6% using simple and accurate time stamp reconstruction algorithm.

On the real devices, we performed the basic and accurate sample time reconstruction for different configured FIFO fill levels in a range from 35 to 350 bytes. The sensor samples are generated with a rate of 200 Hz and each sample takes seven bytes. Hence, the FIFO stream is fetched every 25 to 250 ms, respectively. The readout of the FIFO stream, together with the sensor timer value, is triggered by the fill level interrupt in all tests. The SPI bus clock was driven at 10 MHz in all experiments. Each test case was executed 250 times for about 30 s on both sensors. Figure 7 shows the resulting standard deviation of the sample-to-sample distance of both algorithms for the different FIFO fill levels.

While the first sensor, shown in Figure 7a, has no noticeable drift, the standard deviation of the sample-to-sample distances stay below 80 μs for both time stamp reconstruction methods. Even on a higher fill level of the sensor FIFO, the error is not increasing because the real sampling periods of the first sensor device matches the ideal sampling periods, that are used in the simple time stamp reconstruction algorithm. However, the variation of the results for the simple method is slightly higher due to the uncorrected communication jitter.

In Figure 7b, the second sensor shows a relative drift of 1.6% between the time bases of the raspberry pi and the sensor device. Here, the standard deviation of the sample-to-sample distances and accordingly the jitter of the reconstructed time stamps differ widely between the two presented methods. The mean error of the simple reconstruction algorithm increases steadily using a higher size of the FIFO stream. On the other hand, the ASTR algorithm is able to completely compensate the relative drift and to keep the overall jitter low independently of the FIFO fill level. In this particular case, at a relative drift of 1.6%, the ASTR performs better than the simple approach with factor 20 on a low fill level to 130 on a higher fill level.

**Figure 8:**
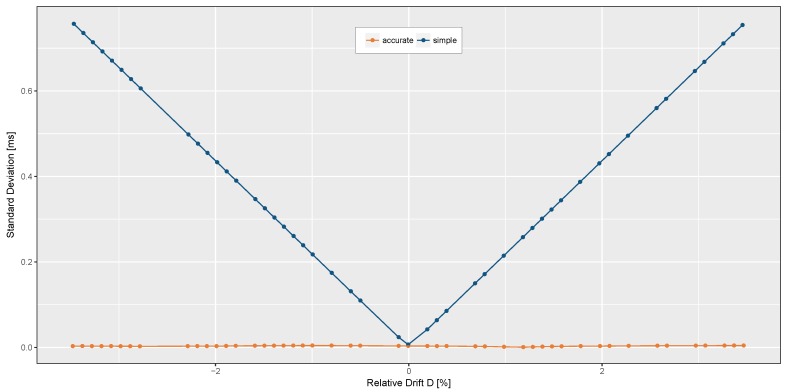
Comparison of simulated sample-to-sample distances for different relative drifts using simple and accurate time stamp reconstruction algorithm.

To prove the independence from the relative drift, a couple of simulations with a randomly varying time base ratio *D* were done. Therefore, the fill level of the FIFO interface was configured to 140 bytes. The data path settings of the simulated sensor were, equally to the real sensor experiments, configured to 200 Hz. Also the length of each test case was limited to 30 s of simulation time. The host-sensor communication was simulated using the SPI protocol running at 10 MHz. In total, 100 simulation runs with random time base ratios *D* in a range of −3.5% to +3.5% have been executed. For the time stamp reconstruction, the same algorithms were used as on the real device. The results are plotted in Figure 8. The simulation results substantiate the assumption that the timing jitter can be almost completely corrected by the presented approach independent of the relative Drift *D*. However, ignoring the timing information of the sensor device leads to a linear rising timing error concerning the absolute relative drift.

Estimating the power consumption of the presented approaches is difficult, as it depends significantly on the underlying system architecture. It will vary to a large extent between an micro-sized embedded sensor hub controller and a more powerful processor of a state-of-the-art mobile phone. Nevertheless, to give an impression of the influence of the sample time reconstruction algorithms onto the power consumption, we measured the run-time of the full measurement and reconstruction setup running on the raspberry pi platform.

**Figure 9:**
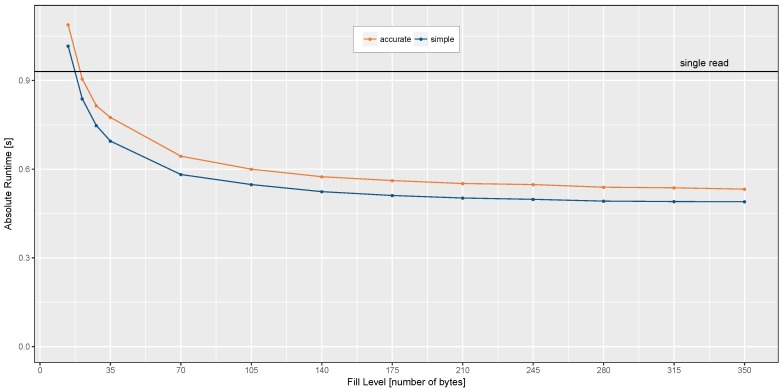
Absolute run-time measurements of both algorithms using different fill levels in comparison with single sample readout, represented by the black line.

We limited all test case executions to 30 s total length and used the time command of the linux bash to measure the active time of the process. For reading the sensor data sample by sample without doing any time stamp reconstruction, the absolute run-time accumulates to 0.93 s, which corresponds to 3.1% processor utilization. Blocking times, waiting for the read access to be finished, are not considered in this number. The results of the absolute run-time measurements are shown in Figure 9. Using a quite small fill level of only two samples allowed the processor utilization slightly increase by 0.5% calculating the ASTR and by 0.2% performing the simple time stamp reconstruction. If the fill level of the FIFO interface is greater than two samples (14 bytes), the absolute run-time becomes smaller than the single read approach. With an increasing fill level the total process utilization approaches 1.8% and 1.6% for the accurate and simple reconstruction algorithm, respectively. Despite the additional processing overhead for decoding the FIFO stream, calculating the relative drift and adjusting the sample time stamps, the overall processor utilization can be lowered by 42% using the FIFO stream based accurate reconstruction approach instead of fetching every sample one-by-one.

## 6. Conclusions

With the increasing number of sensors that are built into mobile phones, fitness trackers, game controllers, clothes and many other mobile devices, the fusion of the locally available sensor data becomes more and more important. However, the acquisition of the data is increasingly time and power consuming on a growing number of connected devices. Data loss and timing inaccuracies due to communication delays make the sensor fusion and feature extraction more difficult. To relax this problem, many of the modern sensors allow the reading of multiple data samples in a FIFO ordering to lower the number of communication attempts and to avoid data loss. However, reading the data as a FIFO stream obligates the host device to reconstruct the temporal order of the single samples to allow an event-synchronized sensor fusion.

In this paper, we presented an algorithm for an accurate reconstruction of the sampling times of each sensor sample in a FIFO stream. Section 4 explains the calculation of the relative drift between the time bases of a sensor device and the host by multiple read accesses to the sensors time information. Furthermore, the same timer can be used to reduce the timing offset error. The shown algorithm is able to lower the signal jitter of FIFO stream data significantly. It can eliminate the influence of relative clock drifts as well as communication latencies between multiple connected sensor devices and the host device and finally align the fetched sensor samples onto one time base. This is done without adjusting the clocks of the sensor devices which makes the solution suitable for all devices offering the internal timer values and a FIFO interface. Thus, the sensor fusion quality can be kept high, while the power consumption of the data processing system can be decreased. Although the FIFO based approaches have a higher complexity compared to a single read and store approach, those calculations as well as the communication attempts to the sensor devices can be executed more rarely. The time and space complexity of the presented algorithm is linear (O(n)).

To prove the accuracy of the presented algorithm, a couple of experiments were done with two BMI160 IMUs [6], as well as simulations on a virtual sensor prototype modeled in SystemC. The results of the ASTR algorithm were compared with the simple FIFO data reconstruction without using the sensor time information. Both, the experiments and the simulation stated a full compensation of the negative influence of relative drift onto the reconstructed sensor sampling times. The standard deviation of the reconstructed sampling period stayed below 40 μs, independent of the FIFO fill level, while the granularity of the sensor timer itself has 39 μs. Additionally, the processor utilization could be lowered by 42% using the FIFO based ASTR with one sensor device on a raspberry pi 2B. As an effect, this can increase the IDLE time of the processor and reduce the power consumption of the mobile platform.

The study showed that the FIFO based sensor data acquisition using ASTR is an advantageous alternative to the currently, often implemented, single sample-based algorithms.

## Figures and Tables

**Table 1:** Sensor timer aligned data rates.

**Bit *m* in Sensor Time**	…	10	9	8	7	6	5	4	3	…
**Resolution [ms]**	…	40	20	10	5	2.5	1.25	0.625	0.3125	…
**Sensor Sample Rate [Hz]**	…	25	50	100	200	400	800	1600	3200	…

## Abbreviations

AR	Augmented Reality
ASTR	Accurate Sample Time Reconstruction
FIFO	First-In-First-Out
I2C	Inter-Integrated Circuit
IMU	Inertial Measurement Unit
LSB	Least Significant Bit(s)
MEMS	Micro-Electro-Mechanical Systems
RBS	Reference Broadcast Synchronization
SPI	Serial Peripheral Interface
TPSN	Timing-Sync Protocol for Sensor Networks
UART	Universal Asynchronous Receiver Transmitter
UTC	Coordinated Universal Time
VR	Virtual Reality
